# Annotations

**Published:** 1887-11-12

**Authors:** 


### Annotations.

The climate of Great Britain does not rouse enthusiasm even in the souls of the most patriotic. It may be true that "Britons never shall be slaves," but it is not certain that they shall never be wet to the skin, or half-dead with catarrh, or groan with rheumatic fever, or die of pleuropneumonia. The French tell us we have most wretched weather, and we cannot truthfully contradict them. No doubt other countries have characteristics of climate which disagree with their inhabitants considerably; as, for example, the centre of Africa, which roasts Europeans alive and makes even the natives uncomfortable. But for all-round miserableness during two or three months in the year commend us to England, and especially to London or other large towns. We have cold without frost, snow without ice, winter without skating, and light without sun. No wonder we are melancholy and grim, and set ourselves to hard work in sheer desperation and because of the impossibility of finding rational pleasures. The "southerly wind and the cloudy sky" may "proclaim it a hunting morning," and thereby put the squire in spirits and bring out a good field; but to the town man the moist and cloudy sky, whether the wind is south or north, means leaden spirits and often wet clothes, dirty boots, and general disgust at all things. Almost the worst enemy Englishmen have, from a health point of view, is their climate. It needs to be thoroughly understood and provided against. The man of large experience acquires the habit of "dodging" the weather. If he can arrange his business so as to stay indoors on very bad days, he will do so; but, if not, he takes care to have varieties of clothing suitable for every season, and for those most difficult of all periods to manage, the transition times when the seasons are changing. Sometimes he will wear an overcoat in June; but no degree of warmth will make him go without one at Christmas, if he is a wise man. The one defence which is of the greatest service to men and women in this country is the constant wearing of woollen underclothing all the year round. At least three different degrees of thickness should be in use; and it should not be considered too much trouble to change from one to the other two or three times a-week, if the weather demand it. A little intelligent forethought of this kind is well repaid in freedom from colds, and much worse ills.

**A Dangerous Climate.**

Many persons of sensitive skin have strong objections to woollen clothing on account of its irritating properties. In some people the sensations produced are so marked as to make the wearing of such clothing impossible. But whilst it is admitted that there may be now and again a person who cannot wear woollen, just as there may be a person in a million who cannot take quinine or opium or oysters, it is nevertheless true that many refuse to wear it for purely fanciful reasons. Of course it may for two or three days cause much irritation and feelings of impatience, but if these be resolutely endured they will be speedily vanquished. Whether woollen cause irritation or not, it is certain that it is of the greatest possible value as clothing in the climate in which English men and women have to live. Some persons have very decided tendencies to rheumatism, and to combat these tendencies nothing is equal to pure woollen garments. Others are subject to colds and various catarrhs on the slightest provocation, and here again woollen is the best defence. Not a few, especially of those who have lived in tropical climates, experience frequent disorders of the liver and spleen with much consequent derangement of the general health. To these, woollen clothing is of the very primest importance, as most of them very well know, and prove the reality of their faith in it by wearing it night and day. Woollen is suitable both for summer and winter wear. Nothing produces more discomfort on a hot summer day than a garment, other than woollen, which is moist with perspiration, and clings like a dripping sheet around the body. But if woollen be worn and frequently changed, the perspiration is all absorbed, and chills are unknown. In winter the advantages of thick and soft wool clothing are so obvious that nothing need be said about them. The general conclusion is this, that every person in Great Britain should wear woollen underclothing in some form; and those who think that personal idiosyncrasy stands in their way should set themselves steadfastly to overcome it; and especially does this apply to those who are approaching old age.

**Under-clothing.**

The *British Medical Journal* has done excellent service by calling the attention of the public to certain home products which have been to some extent put into the background lately. The system of underclothing which goes by the name of Dr. Jaeger, has been in all men's mouths, and in all men's wardrobes as well. We have not a word to say against it. On the contrary, the system is good, and so are the garments. The success of the business shows how very much may be accomplished in a short time, by the clever application of means to ends. Dr. Jaeger believes in woollen "from top to toe;" and he sets about making all kinds of woollen clothing, of all degrees of stoutness and softness, to suit all purses and all tastes; even going so far as to make gloves for the feet. That done, he proceeds to make known by liberal advertisement the great advantages of his system; and this he does with so much judgment and energy, that very soon his marts overflow with business and his coffers with gold. But what does it all mean? Has Dr. Jaeger invented a new kind of sheep, which produces a softer and warmer kind of wool? Or has he discovered some method of manipulation, which converts the wool of the common sheep into an article superior to that produced by ordinary processes? He has done neither. He has simply carried out the system of woollen underclothing thoroughly, instead of partially, literally clothing the wearer "from top to toe," toe included. And, then having done the thing thoroughly, he has proclaimed it thoroughly from every housetop, as all the world knows. He has reaped his reward, as he well deserved to do. But our own manufacturers are beginning to cry out that their woollen is as good as Dr. Jaeger's, and better; that their methods of manufacture are as good as his, and much better. Having taken the trouble to inspect and to wear both home productions and Jaeger's, we are able to say with all confidence that the woollen goods of England, Scotland, and Ireland can equal the best of Jaeger's in quality. If our manufacturers will ive to their customers the same variety as Jaeger, and to their goods the same scientific study and publicity, there need be no doubt that purchasers will come back to them. England, Scotland, and Ireland have long been famous all the world over for their woollen goods, and it cannot be doubted that they produce the very best articles in the world's markets. Just now Mr. and Mrs. Ernest Hart are striving to bring Irish woollen underclothing into public notice. Too much credit cannot be given them for their self-denying and persistent efforts to aid the poor Donegal operatives to earn a decent livelihood. Society, too, owes them a debt for providing novel clothing of great beauty and perfect texture. The Donegal Industrial Company, at 43, Wigmore Street, are supplying the clothing of the very finest quality and manufacture, clothing which is woven in the hand loom by Irish peasants, and which cannot be surpassed by any woollen garments in the world. Neither patriotism nor any other feeling would induce us to try to exalt one man by putting down another, but when our own people can supply us with goods of as high quality—or even higher than are supplied elsewhere, and at prices as moderate and reasonable—we cannot help feeling that they too should have some of the plums which enrich the commercial pudding.

